# Gender similarities and differences in the perception of caring among nurses during the COVID-19 pandemic: a mixed-methods study

**DOI:** 10.1186/s12912-023-01267-z

**Published:** 2023-04-12

**Authors:** Lai Kun Tong, Ming Xia Zhu, Si Chen Wang, Pak Leng Cheong, Iat Kio Van

**Affiliations:** Kiang Wu Nursing College of Macau, Macao, China

**Keywords:** Caring, Nurse, Gender, COVID-19 pandemic

## Abstract

**Background:**

Caring is the essence and core of nursing. Current studies on the effect of gender on nurses’ perception of caring have been inconsistent. Most of these studies were quantitative and conducted before the COVID-19 pandemic. The study aimed to explore the gender similarities and differences in nurses’ perception of caring during the COVID-19 pandemic.

**Methods:**

In 2020, a cross-sectional mixed-method study was conducted in 11 cities in China using structured online questionnaire (n = 8030) and face-to-face individual interview (n = 42). Data collection tools included the Caring Dimensions Inventory and semi-structured interview.

**Results:**

In stepwise multivariable linear regression analysis, female nurses scored 1.93 points higher on caring than male nurses, after completely controlling for the other factors. The interview results showed that both male and female nurses agreed that caring was to deal with three kinds of relationships, namely nurses and people, nurses and themselves, nurses and society. There were gender differences in the connotation of caring between nurses and themselves, but not in the connotations of caring between other relationships. The ranking of the importance of caring connotations among these relationships showed no gender difference except between nurses and themselves.

**Conclusion:**

The quantitative findings suggest that gender influences nurses’ perception of caring. The qualitative findings demonstrate that nurses regard themselves as both recipients and implementers of caring. The qualitative results indicate nurses of different genders have similarities and differences in their perception of caring, and the biggest difference lies in the relationship between nurses and themselves.

## Background

Caring is the essence and core of nursing, and caring behavior is considered an integral part of nurses’ professional identity [1]. Specifically, caring describes the professional behavior characteristics of nurses in nursing practice, including their cultural, emotional, moral, interactive and helpful nature [2, 3]. Caring is the accumulation of knowledge and experience and the cultivation of ability [1, 2]. Therefore, caring is an important part of nursing practice and a competence that nurses should have. Evidence shows that caring has positive effects on both patients and nurses [4], such as improved patient satisfaction with care [5, 6] and improved job satisfaction and engagement for nurses [7]. Culture is an important factor affecting the cognition and practice of caring [8, 9]. That is, people from different cultures have different definitions of caring. For example, a study showed that while there were similarities in perceptions of caring between Slovene, Croatian, Chinese, and Russian nursing students, the four countries’ nursing students placed different emphasis on various aspects of caring [10]. Many scholars have discussed the caring in the Chinese context [3, 11, 12]. Chinese nurses considered that caring included caring attitude, caring knowledge, caring ability and caring perception, among which caring attitude was the core [3].

While caring is culturally cultivated, it is gender framed. Nursing has widely been seen as a female occupation. The conflict between masculinity and caring concept has traditionally resulted in female nurses displaying more caring than male nurses [13]. However, more and more men are joining the occupation -- for example, about a quarter of the Dutch nurses are male [14]. Current studies on the effect of gender on nurses’ perception of caring have been inconsistent [15, 16]. There was no significant difference in nurses’ perception of caring between genders in China [15], Iran [17], and Turkey [16], while there was a significant difference in Saudi Arabia [18] and Greece [19]. Most of these studies were quantitative and conducted before the COVID-19 pandemic. The COVID-19 pandemic is affecting nurses’ understanding of their role, as well as the patterns they practice and care for patients [20, 21]. Nurses’ limited knowledge of the disease restricted their ability to practice caring during the early days of the COVID-19 pandemic [3]. COVID-19 pandemic has a negative impact on nurses’ physical and mental health [22], and they are further restricted in their caring practice due to a lack of resources. Nurses have gained a new perspective on caring as a result of the COVID-19 pandemic, in which their professional value [23] and social image [24] have been enhanced. A study of nurses’ perceptions of caring during the COVID-19 pandemic has not yet been conducted. Therefore, in order to fill the research gap, and clarify the gender similarities and differences in nurses’ perception of caring in the Chinese context during the COVID-19 pandemic, a mixed approach was used in this study.

## Methods

### Study design

A mixed method design was adopted, consisting a survey in quantitative phase and one-on-one semi-structured interviews in qualitative phase. The questionnaire-based quantitative phase was conducted from 19 May to 7 August 2020, followed by the qualitative one from 3 July to 28 December 2020.

### Study setting

The study was conducted in 11 Chinese cities, including Dongguan, Foshan, Guangzhou, Hong Kong, Huizhou, Jiangmen, Macao, Shenzhen, Zhaoqing, Zhongshan and Zhuhai. In 2019, these 11 cities had a permanent population of 72.7 million (5.2% of the total population in China) and 258,000 nurses (5.8% of the total number of nurses in China) [25].

### Study sample

#### Quantitative sample

Nurses from the above 11 cities who met the following inclusion and exclusion conditions were recruited. The conditions for inclusion were as follows: (1) Nurses working in hospitals, clinics, schools, elderly services, nursing homes and other health-related institutions. (2) Nurses who can read and write Chinese. The exclusions were as follows: 1) Newly graduated nurses who had not passed the probation period, because these nurses were not able to work independently.

The sample size was estimated using the formula [26] n = z^2^p(1-p)/d^2^, where the z-value of the 95% confidence level was 1.96, d was the permissible error (in this study it was 0.4), p was the percentage occurrence of a state (in this study, 50% was used as an estimate of p to produce the maximum sample size). The minimum sample size of each city was calculated to be 601, so the minimum sample size of this study was calculated to be 6,611. The sample size was increased by 10% due to an estimated response rate of 90%, so the sample size was 7,346.

#### Qualitative sample

Nurses who had completed the questionnaire were considered for the interview. Recruitment of three to eight nurses in each city would be sufficient to achieve data saturation based on previous experience and literature [27]. The research team worked with nursing professional bodies, hospital nursing departments, and nursing colleges in each city to use purposive sampling to identify respondents, taking into account their gender, age, education, work experience, and affiliation to ensure that they were representative. Research team members contacted qualified nurses whose participation was tentatively confirmed by the above-mentioned institutions. Finally, forty-two nurses participated in the semi-structured in-depth interviews.

### Instruments

#### Quantitative measures

The online questionnaire consisted of two parts: general information and the Caring Dimensions Inventory (CDI). The first part included questions about the socio-demographic (such as gender, age, marital status, etc.), profession- and workplace-related characteristics (such as professional title, professional experience, etc.) of the participants. The CDI was developed by Watson et al. [28], and translated into Chinese by Tong et al. [29]. The CDI is a 5-point Likert scale (1 = strongly disagree, 5 = strongly agree) designed to measure the perception of caring. The total score ranges from 25 to 125 and is calculated as the sum of the scores for the 25 items. The scale has three dimensions: nurturance, collaboration, and skill. The dimension score is the average score of items, with higher score indicating more highly endorsed. The Cronbach’s alpha of the CDI was 0.97 [29].

#### Qualitative measures

The research team developed the interview outline based on literature and experience, and then modified the interview outline according to the suggestions of two nursing experts. The outline was further tested by three interviews with qualified participants. Minor revisions were made and a relatively stable interview guideline was formed. Major questions contained in the guideline included: (1) What do you think caring means? Can you give me an example? (2) How do you think caring is implemented in your workplace or other settings? Can you give me some examples? (3) What can be done to improve caring in your organization? (4) Has your understanding of caring changed since graduation? (If yes, continue to ask what has changed) (5) Do you have anything to add to this topic? Interviews lasted between 20 and 40 min.

Data collection and ethical considerations.

Data collection was initiated after ethical approval had been obtained from the authors’ organization (Ref. 2019APR01). The research team partnered with nursing professional bodies, hospital nursing departments, and nursing colleges in 11 cities to promote the project in their cities. The research team produced an electronic publicity poster, and the respondents could access the electronic questionnaire through the QR code or URL on the poster. The research team set the questionnaire platform so that each device could only fill in the questionnaire once, so as to avoid repeated participation in the survey. Before filling in the electronic questionnaire, the respondents must read the informed consent and click the consent button to start. The questionnaire was anonymous and the participants could quit at any time. Participants were not paid for their participation in the survey.

The study was conducted during the COVID-19 pandemic, and the team had originally planned to conduct interviews using online conferencing software or over the phone for quarantine purposes. The above methods were used to conduct the three interviews in three cities in testing the interview outline. Given that the theme of the study was caring, and unstable network could impact the quality of the interviews, face-to-face interviews were conducted after the test stage by a registered nurse trained in interviewing skills. A member of the research team participated in the interviewing by telephone and occupationally asked questions to the interviewees. Before the interview, the interviewer introduced to the interviewee the research background, purpose, interview content, and informed him or her that the interview would be recorded and another interviewer would attend the interview by phone. Participants could terminate the interview or withdraw from the study at any time. Each participant received $100 in local currency (about US $15) as recompense for his or her time to participate in the interview.

### Data analysis

#### Quantitative data analysis

Categorical data were presented as frequency (percentage), and continuous data were expressed as mean (standard deviation). Univariate and multivariate linear regression models were employed to examine predictors of the CDI score. Variance inflation factor (VIF) was used to detect the multicollinearity problem among independent variables. A VIF value above 10 indicates problematic multicollinearity [30]. Variables with a VIF > 10 were excluded to avoid multicollinearity. SPSS 26.0 software was used for statistical analysis. Values of p < 0.05 were considered statistically significant.

#### Qualitative data analysis

The digital audio recordings of the interviews were transcribed verbatim in Chinese by trained professionals, and the research team checked the transcriptions for accuracy. The data analysis was based on Chinese transcripts. Translations of the Chinese transcriptions were conducted by the research team and were limited to segments relating to the presentation of the research results in this article. The translations were cross-checked by a bilingual colleague, who was not in the research team, to ensure accuracy.

Ritchie and Spencer’s Framework approach [31] was used to analyze the qualitative data. The first author and the corresponding author worked out a code book after the two read through the interviews for several times. When the code book became stable after sufficient discussions between the two, they independently coded all the interviews. Any discrepancy was resolved through discussion with the second author. The main findings were reported to other members of the research team and the team discussed to finalize the themes of the data. The transcript data were managed and coded using Nvivo 12.0. NVivo matrix queries were conducted to highlight similarities and differences across gender. The coded data were inserted into a matrix with each row for a subtheme and each column for a gender, which charted the themes against each gender.

## Results

### Quantitative findings

#### Characteristics of participants

A total of 8,030 valid questionnaires were obtained, including 274 (3.4%) from men and 7,756 (96.6%) from women. Most of the participants were married (63.9%), had children (59.1%), graduated from college (52.8%), and worked in hospitals (97.6%). Male participants’ age, proportion of married, proportion of having children, professional title and working years in nursing profession were lower than female participants (p < 0.05), while their educational background was higher than female participants (p < 0.05) (Table 1).

**Table 1:** Characteristics of Participants in Quantitative Study

Characteristics		Categories	Combine (N = 8030) n (%)	Male (N = 274) n (%)	Female (N = 7756) n (%)	χ^2^ / t	p
*Socio-demographic characteristics*							
Age ^a^			31.9 (8.7)	28.7 (6.7)	32.0 (8.8)	-7.901	< 0.001
Marital status						46.985	< 0.001
		Single	2741 (34.1)	146 (53.3)	2741 (34.1)		
		Married	5127 (63.9)	126 (46.0)	5127 (63.8)		
		Other	162 (2.0)	2 (0.7)	162 (2.0)		
Had one or more children						52.618	< 0.001
		Yes	4748 (59.1)	104 (38.0)	4644 (59.9)		
		No	3282 (40.9)	170 (62.0)	3112 (40.1)		
Educational level						10.644	0.005
		Diploma	3654 (45.5)	112 (40.9)	3542 (45.7)		
		Graduate	4240 (52.8)	151 (55.1)	4089 (52.7)		
		Postgraduate	136 (1.7)	11 (4.0)	125 (1.6)		
*Profession related characteristics*							
Professional title						17.976	< 0.001
		Junior	5542 (69.0)	221 (80.7)	5321 (68.6)		
		Intermediate and senior	2488 (31.0)	53 (19.3)	2435 (31.4)		
Administrative supervisor						0.004	0.950
		Yes	918 (11.4)	31 (11.3)	887 (11.4)		
		No	7112 (88.6)	243 (88.7)	6869 (88.6)		
Received caring training in college						1.408	0.495
		Yes	6748 (84.0)	237 (86.5)	6511 (83.9)		
		No	869 (10.8)	24 (8.8)	845 (10.9)		
		Not sure	413 (5.2)	13 (4.7)	400 (5.2)		
Year in nursing profession ^a^			11.1 (8.5)	6.8 (5.8)	11.3 (8.5)	-12.490	< 0.001
Year in present organization ^a^			9.1 (7.5)	5.8 (4.9)	9.3 (7.6)	-11.278	< 0.001
The number of caring training in the most recent year ^a^			1.4 (2.1)	1.4 (1.8)	1.4 (2.1)	-0.423	0.672
*Workplace related characteristics*							
Type of health facility						1.741	0.187
		Hospital	7834 (97.6)	264 (96.4)	7570 (97.6)		
		Community	196 (2.4)	10 (3.6)	186 (2.4)		
Human resource						2.865	0.239
		Enough	2021 (25.0)	57 (20.8)	1955 (25.2)		
		Barely enough	3146 (39.2)	116 (42.3)	3030 (39.1)		
		Not enough	2872 (35.8)	101 (36.9)	2771 (35.7)		
Received caring training provided by employers						2.737	0.255
		Yes	5981 (74.5)	197 (71.9)	5784 (74.6)		
		No	1298 (16.2)	54 (19.7)	1244 (16.0)		
		Not sure	751 (9.3)	23 (8.4)	728 (9.4)		
CDI							
	Total score ^a^		107.6 (14.7)	105.3 (17.5)	107.6 (14.6)	-2.480	0.013
	Nurturance ^a, b^		4.4 (0.6)	4.30 (0.72)	4.38 (0.59)	-2.241	0.025
	Collaboration ^a, b^		4.2 (0.6)	4.14 (0.73)	4.23 (0.64)	-2.404	0.016
	Skill ^a, b^		4.2 (0.6)	4.15 (0.74)	4.23 (0.64)	-2.134	0.034

Notes: ^a^, result from mean (SD) and t-test; ^b^, items mean score; CDI, the Caring Dimensions Inventory

#### The impact of gender on the perception of caring

The CDI score was higher for female than male (107.6 vs. 105.3, p = 0.013). Among the three dimensions, the Nurturance dimension was endorsed the most by both males and females (Table 1). The average score of female participants in all three dimensions was higher than that of male participants (Table 1). In stepwise multivariable linear regression analysis, gender (female vs. male) (p = 0.031), marital status (married vs. unmarried) (p = 0.019), administrative supervisor (no vs. yes) (p = 0.002), number of caring training in the most recent year (p < 0.001), type of working institution (community vs. hospital) (p = 0.028), and human resources of working institution (barely enough vs. enough) (p = 0.010) (insufficient vs. sufficient) (p < 0.001) and the availability of caring training by work institution (no vs. yes) (p < 0.001) (unclear vs. yes) (p < 0.001) were considered independent predictors of CDI score (Table 2). All variables included in the multivariable linear regression model did not show multicollinearity (VIF: 1.01–1.59). Female nurses scored 1.93 points higher on CDI than male nurses, after completely controlling for the other factors (Table 2).

**Table 2:** Association of the Caring Dimension Inventory Score

Variables (Reference)		Univariate analysis		Multivariate analysis		
		β (95%CI)	p	β (95%CI)	p	VIF
*Socio-demographic characteristics*						
Gender (Male)						
	Female	2.24 (0.47, 4.00)	0.013*	1.93 (0.18, 3.67)	0.031*	1.01
Age		0.02 (-0.02, 0.06)	0.25	/		
Marital status (Single)						
	Married	0.76 (0.08, 1.44)	0.03*	0.81 (0.13, 1.49)	0.019*	1.07
	Other	-0.13 (-2.46, 2.19)	0.91	/		
Had one or more children (No)						
	Yes	0.60 (-0.06, 1.25)	0.074	/		
Educational level (Diploma)						
	Graduate	0.74 (0.09, 1.39)	0.025*	/		
	Postgraduate	1.21 (-1.30, 3.73)	0.344	/		
*Profession related characteristics*						
Professional title (Junior)						
	Intermediate and senior	0.98 (0.29, 1.68)	0.006*	/		
Administrative supervisor (Yes)						
	No	-1.72 (-2.72, -0.71)	0.001*	-1.62 (-2.65, -0.59)	0.002*	1.07
Received caring training in college (Yes)						
	No	-1.85 (-2.89, -0.82)	< 0.001**	-1.57 (-2.64, -0.51)	0.004*	
	Not sure	-4.70 (-6.15, -3.24)	< 0.001**	-3.44 (-4.91, -1.97)	< 0.001**	1.05
Year in nursing profession		0.02 (-0.02, 0.06)	0.353	/		
Year in present organization		0.03 (-0.02, 0.07)	0.225	/		
The number of caring training in the most recent year		0.68 (0.53, 0.83)	< 0.001**	0.44 (0.29, 0.60)	< 0.001**	1.10
*Workplace related characteristics*						
Type of health facility (Hospital)						
	Community	-3.67 (-5.75, -1.59)	0.001*	-2.33 (-4.40, -0.25)	0.028*	1.03
Human resource (Enough)						
	Barely enough	-1.30 (-2.12, -0.48)	0.002*	-1.06 (-1.87, -0.25)	0.010*	1.57
	Not enough	-2.97 (-3.80, -2.14)	< 0.001**	-2.38 (-3.21, -1.54)	< 0.001**	1.59
Received caring training provided by employers (Yes)						
	No	-4.55 (-5.43, -3.68)	< 0.001**	-3.14 (-4.06, -2.22)	< 0.001**	1.16
	Not sure	-4.10 (-5.21, -3.00)	< 0.001**	-2.55 (-3.70, -1.41)	< 0.001**	1.11

Notes: /, variable was excluded from the regression model

### Qualitative findings

#### Characteristics of participants

A total of 42 nurses were interviewed, including 11 males and 31 females. The demographic data of interviewees and questionnaire participants were similar. Male participants’ age, proportion of married, and working years in nursing profession were lower than female participants (Table 3).

**Table 3:** Characteristics of Participants in Qualitative Study

Characteristics	Categories	Combine (N = 42) n (%)	Male (N = 11) n (%)	Female (N = 31) n (%)
Age ^a^		36.1	29.5	38.5
Marital status				
	Single	17 (40.5)	5 (45.5)	12 (38.7)
	Married	24 (57.1)	6 (54.5)	18 (58.1)
	Other	1 (2.4)	0 (0.0)	1 (3.2)
Had one or more children				
	Yes	18 (42.9)	5 (45.5)	13 (41.9)
	No	24 (57.1)	6 (54.5)	18 (58.1)
Educational level				
	Diploma	7 (16.7)	2 (18.2)	5 (16.1)
	Graduate	29 (69.0)	9 (81.8)	20 (64.5)
	Postgraduate	6 (14.3)	0 (0.0)	6 (19.4)
Year in nursing profession ^a^		14.2	7.2	16.7

Notes: ^a^, result from mean

#### Gender similarities and differences in the perception of caring

The interview results showed that both male and female nurses agreed that caring was to deal with three kinds of relationships, and the most mentioned relationships of different genders were in the same order, namely nurses and people, nurses and themselves, nurses and society (Table 4). However, nurses of different genders did not perceive the connotations of these three relationships in the same way.

**Table 4:** Emerged Themes from the Interviews

Themes	Sub-Themes	Female n (%)	Male n (%)	Sample responses
**Nurse and People**	Holistic care	31 (100.0)	11 (100.0)	“When we treat patients in the ICU (Intensive Care Unit), we often make a personalized care plan for patients according to their illness, family background, and even economic conditions, and then carry out targeted care measures for patients.” (30-year-old male nurse)
	Mutual respect	25 (80.6)	9 (81.8)	“This is a process of mutual respect. […] It makes you feel good when someone is really nice, and you want to do more for them.” (26-year-old female nurse)
	Shared decision making	6 (19.4)	1 (9.1)	“We (the medical team and the patient) should discuss it and they (the patient) have a choice as to the kind of medical care they want.“ (37-year-old female nurse)
**Nurse and themselves**	Professional pursuit	18 (58.1)	4 (36.4)	“You know there’s no end to learning. […] You need to keep learning so that you can cope with things.” (24-year-old male nurse)
	Self-acceptance	12 (38.7)	0 (0.0)	While I usually get along with everyone, I tend to be more rational and my perception ability is not as strong [.] leading to a lot of things patients find painful, and I can’t give them much support. (33-year-old female nurse)
	Staying true to original aspiration	7 (22.6)	3 (27.3)	“There was a fire in our soul upon graduation. […] But there is a growing sense that there is no such thing as a possibility, or that even if you try too hard, it won’t work.” (31-year-old female nurse)
	Professional identity	0 (0.0)	1 (9.1)	“In addition to the things doctors can do to help patients, we can also provide them with things that are useful.“ (24-year-old male nurse)
**Nurse and society**	Positive practice environment	14 (45.2)	5 (45.5)	“Obviously, the nursing workforce is very large, but not very vocal, right? […] That means there might be some problems.” (36-year-old male nurse)
	Social responsibility	7 (22.6)	1 (9.1)	“Go where there is epidemic, fight it till it perishes. […] Everyone in our department cancelled the Spring Festival holiday and was on standby at work.” (23-year-old male nurse)

#### Nurses and people

Nurses of both genders agreed that clients they served included patients and their families. They identified three themes on the relationships between nurses and their patients/families, and both male and female nurses ranked the themes in the same order of importance: holistic care (100% vs. 100%), mutual respect (80.6% vs. 81.6%) and shared decision making (19.4% vs. 9.1%), but female nurses tend to emphasize shared decision making more often.

Holistic care was client-centered and provided physical, psychological, social and spiritual care. It was regarded by the nurses as the essential component of caring. Both male and female nurses seemed proud that they had tried their best to meet the various needs of their patients in their daily work practice. Mutual respect was emphasized by both males and females because it was inherent in holistic care. The nurses elaborated that the nurse-patient relationship has transformed from paternalism into partnership, and the nurse-patient relationship should be changed from active - passive model, guidance - cooperation model to mutual participation model. Patients should have a say in the decision making of nursing plans, “Work with him (patient) on a care plan. […] Only he knows his own situation best. […] After making the plan, work on it together.” (45-year-old female nurse).

#### Nurses and themselves

Three themes emerged related to nurses’ self-identity as a nurse, and the content and order of importance of these themes varied by gender. For male nurses, the three themes in descending order of importance were professional pursuit (58.1%), staying true to original aspiration (38.7%), and professional identity (22.6%), while for female nurses, they were professional pursuit (36.4%), self-acceptance (27.3%), and staying true to original aspiration (9.1%).

Caring was a kind of ability. The nurses in the study reflected the importance of continuous learning to refine their professional skills. Some of them extended reading nursing books into extensive reading as reading on a wide range of books could improve their inner cultivation, “I like reading books. This is the inner cultivation. Reading can improve our ability, our own inner cultivation. […] And then to better serve others.” (33-year-old female nurse). The original intention that most nurses joined the nursing profession was their desire to help others. Nurses are constantly self-reminded of sticking to their original desire in case their passion of helping others might fade with time, “You can continue to do nursing passionately if you remain interested in the patient.” (24-year-old male nurse).

Professional identity was a theme unique to male nurses. Male nurses saw professional identity as part of caring. As the implementers of caring, nurses needed to admit the significance and value of the nursing profession and affirm their professional ability, so as to give full play to the advantages of the nursing profession and effectively implement caring, “We (nurses) can do something to help patients. […] Before patients see the doctor, we assess them and make a preliminary judge on the problems the patients may have from our professional point of view and provide them with other ways to deal with the problems in addition to medications.” (24-year-old male nurse).

Self-acceptance was a theme unique to female nurses. Female nurses in the study confessed that, as human beings, nursing professionals should recognize and accept their own strengths and weaknesses. Some of the experienced nurses recounted the importance of self-reflection in self-acceptance of own weaknesses, “In the first few years of working. […] I thought I had good skills. […] I thought I was valuable. But over time […] In recent years, I felt like I couldn’t get into other people’s hearts, so I felt a little frustrated. […] I become more rational […] I’m used to a lot of things that patients find painful so I can’t give them much support.” (33-year-old female nurse).

#### Nurse and society

Two themes emerged associated with the relationship between nurses and society, and both male and female nurses placed the same order of importance on these two themes: positive practice environment (45.2% vs. 45.1%) and social responsibility (22.6% vs. 9.1%).

Nurses believed that a positive social environment is essential to them for carrying out caring, which included equal opportunities for professional development of nurses in different health institutions, supportive policies, empowered nursing professional bodies, etc. They particularly pointed out that the media should strengthen the comprehensive portrayal of the nursing profession so that the public understands nurses objectively.

While nurses called for positive social environment they were clear of their responsibilities as professionals. They particularly had a sense of responsibilities during social crisis, for example, under COVID-19 pandemic, “Our facility is a drug treatment center (methadone) and it’s year-round. […] (During COVID-19,) office buildings were closed and social workers were not required to provide services, but we, as frontline nurses, maintained services.” (45-year-old female nurse).

## Discussion

To our knowledge, this study is the first to employ a mixed method to explore gender similarities and differences in nurses’ perception of caring. The quantitative analysis indicates that female nurses had higher CDI scores than male nurses, after completely controlling for the other factors. The qualitative findings suggest that nurses in both genders view caring as nurses give caring to clients, themselves, and society while receiving it from these entities. The connotation of caring between nurses and clients, between nurses and society did not differ with the gender of nurses. The ranking of the importance of caring connotations among the above two relationships showed no gender difference. There were gender differences in the connotation of caring between nurses and themselves.

Both quantitative and qualitative results of this study showed that although male and female nurses had different perceptions of caring, there were similarities. Previous studies and theories on caring have emphasized the nurse-patient relationship [32, 33], and our results showed that the nurses interviewed rated the nurse-people relationship as the most important. Both male and female nurses ranked the sub-themes of nurse-people relationship in the same order of importance: holistic care, mutual respect and shared decision making. This indicates that nurses of different genders perceive caring as recognizing and respecting the uniqueness and value of each individual. Our findings are consistent with other studies. Caring requires empathy [34], as well as acceptance and respect [35]. Leininger’s cultural care theory proposed that nurses should provide clients with nursing measures that are integrated with their cultural values, beliefs and lifestyles [36]. The sub-themes of nurse-people relationship were ranked equally by female and male nurses, but female nurses tend to make more statements about shared decision-making. It may be related to gender differences in communication styles and leadership styles [37]. Female nurses tend to perform more partnership-building, take fewer directives and take a more collaborative approach to decision-making than male nurses [38]. On the other hand, male nurses are more individualistic [38]. However, the results of this study are also different from previous studies. Previous studies on caring between nurses and clients mainly put forward requirements for nurses, while this study proposes that caring includes mutual respect between nurses and clients. Nurses are not only the implementers of caring but also the recipients of caring.

In addition to the nurse-people relationship, the results of this study showed that caring also involves dealing with the relationship between nurse and themselves, nurse and society. Nurses of different genders had similar views on nurse-society relationship. A positive practice environment was considered by both male and female nurses to be the most important sub-theme in dealing with the nurse-society relationship. This may be due to the fact that our study was conducted during the COVID-19 pandemic. During the crisis, about a third of nurses suffered from psychological symptoms [39] and about half of all COVID-19 positive health care workers were nurses [40]. In other words, nurses’ physical and mental health is at risk as never before during the COVID-19 pandemic. Academics around the world are calling for attention to the well-being and resilience of frontline nurses [41], providing them with emotional support and protection [42], and providing them with proper working equipment [43, 44]. However, the epidemic has not been contained, and most medical facilities have not put forward good solutions to the above problems. This causes nurses to face clinical ethical problems related to caring when providing services to patients [44]. In non-pandemic time, nurses indicated that organizational factors such as workload, staffing issues, shift work, and lack of management support were significant barriers to practice caring [45]. The COVID-19 pandemic has made these problems worse, but the nurses interviewed stated that in the face of the pandemic and the difficult situation it created, the nurses’ sense of social responsibility helped them overcome fear, stick to their duties and cope with the difficulties. This finding is in line with other studies [46]. COVID-19 has had an impact on nurses’ perceptions of caring, and nurses look forward to a positive practice environment during the crisis. More research is needed to understand nurses’ perceptions of caring in the new context as COVID-19 continues and public health emergencies of international concern become more frequent.

Although nurses of different genders had the same views on caring, they were not completely consistent. The qualitative results demonstrated that the order of importance of themes varied by gender, even though the themes were the same. The results of the quantitative study showed that there was still a difference in CDI scores between male and female nurses after adjusting for other factors. But this finding is not in line with some previous studies [47, 48] revealing no significant gender differences in perception of caring. The theoretical basis for nursing care is the same for nurses regardless of their gender. As a result of this theoretical preparation, variations caused by gender can be avoided in the perception and practice of caring [47]. In addition to cultural factors, the reasons for the inconsistent results may also be influenced by the gender ratio of the participants. Approximately 3% of this study’s participants were male, compared with more than 20% in other studies. The proportion of registered male nurses in China was about 2% in 2017 [49], which was extremely low compared to developed countries. Similarly, the proportion of male nursing students in China is extremely low, resulting in limited educational opportunities for them, such as being marginalized by female by female counterparts in group tasks [50]. Nursing educators need to adopt strategies to integrate male nursing students into predominantly female classrooms. Despite the fact that men may be limited by their innate temperament, which makes them weak in caring, nursing educators can make full use of their innate critical thinking, as research suggests that critical thinking is a potential intervention element in caring [51].

Caring for oneself makes it possible to transfer the experience of caring from oneself to others [3, 52]. In this study, the main difference between male nurses and female nurses in their perception on caring lies in dealing with the relationship between nurses and themselves. The results of this study indicate that professional identity is unique to the perception of caring held by male nurses, whereas self-acceptance is unique to that of female nurses. In studies of self-concept and professional identity development among nurses, caring was identified as the most influential factor [53]. Studies demonstrated that nurses saw caring as an integral part of their professional identity [53]. The perception of caring of both male and female nurses is supposed to include self-acceptance and professional identity, but why are the two so different? This may be because male nurses are more influenced by extrinsic factors while female nurses are more influenced by intrinsic factors [54]. Women choose a career in nursing because they think their personality is suitable for the profession, while men choose it for economic security [55]. However, the reasons for this difference need to be confirmed by further research.

The findings of this study showed that nurses who had received caring training in college and their employers provided caring training had a higher perception level of caring, and the more times they received caring training in the past year, the higher their perception level of caring. It suggests that the availability of caring training for nurses in nursing education institutions and medical institutions can improve their perception of caring. However, this study found that participants had received caring training less than twice less than twice in the past year. Chinese nurses were overworked during the COVID-19 pandemic, making it difficult to take part in caring training [56]. Previous studies indicate that the caring culture of institutions influence nurses’ capability to provide caring [8]. Furthermore, a positive organizational culture can motivate nurses to provide caring by strengthening their sense of meaning at work [3, 57]. Hence, medical institutions can develop an organizational culture that promotes caring so that nurses who cannot receive caring training still maintain their caring perception level.

The present study has led to a better understanding of gender similarities and differences in the perception of caring among Chinese nurses, but it is not without limitations. Firstly, non-randomized sampling might lead to selection bias and reduces the generalizability of our findings. Secondly, the interviewees were selected by purposive sampling, which is prone to researcher bias. Thirdly, although the research team established sampling criteria after repeated discussions, the information contained in the criteria may not be accurate enough to reflect factors that influence nurses’ perceptions of caring and there were chances that better informed participants were overlooked. Fourth, these 11 Chinese cities involved in the study have different medical environments (such as medical systems, medical resources, and caring cultures), which may affect nurses’ perceptions of caring. However, this study did not account for this factor, so it can be discussed in future studies.

## Conclusions

This mixed methods study provides new insights into the differences and similarities of caring perceived by nurses of different genders. The quantitative findings suggest that gender influences nurses’ perception of caring. The qualitative results indicate nurses of different genders have similarities and differences in their perception of caring, and the biggest difference lies in the relationship between nurses and themselves. More research is needed to understand nurses’ perceptions of caring in the new context as COVID-19 continues and public health emergencies of international concern become more frequent. As the proportion of male nurses in China increases, more male nurses’ voices should be added in future studies to further explore whether there are differences in the views of nurses of different genders on caring.

## Data Availability

The data that support the findings of this study are available from the corresponding author, upon reasonable request.

## References

[1] Sebrant L, Jong M (2021). What’s the meaning of the concept of caring?: a meta-synthesis. Scand J Caring Sci.

[2] Taghinezhad F, Mohammadi E, Khademi M, Kazemnejad A (2022). Humanistic care in nursing: Concept Analysis using Rodgers’ Evolutionary Approach. Iran J Nurs midwifery Res.

[3] Tong L-K, Zhu M-X, Wang S-C, Cheong P-L, Van I-K (2022). Factors influencing caring behaviour among registered nurses during the COVID-19 pandemic in China: a qualitative study using the COM-B framework. J Nurs Adm Manag.

[4] Holly W, Fazzone PA, Sitzman K, Hardin SR (2019). The current intervention studies based on Watson’s theory of human caring: a systematic review. Int J Hum Caring.

[5] Kibret H, Tadesse B, Debella A, Degefa M, Regassa LD (2022). The Association of Nurses Caring Behavior with the level of patient satisfaction, Harari Region, Eastern Ethiopia. Nursing: Res Reviews.

[6] Labrague LJ, De los Santos JAA, Tsaras K, Galabay JR, Falguera CC, Rosales RA (2020). The association of nurse caring behaviours on missed nursing care, adverse patient events and perceived quality of care: a cross-sectional study. J Nurs Adm Manag.

[7] Zamaniniya Z, Khademi M, Toulabi T, Zarea K (2021). The outcomes of humanistic nursing for critical care nurses: a qualitative study. Nurs Midwifery Stud.

[8] Albaqawi HM, Alquwez N, Almazan JU, Alharbi SM, Catimbang CC, Rivera PP et al. Workplace Spiritual Climate and Its Influence on Nurses’ Provision of Spiritual Care in Multicultural Hospitals. Religions. 2019;10(2).

[9] Oluma A, Abadiga M (2020). Caring behavior and associated factors among nurses working in Jimma University specialized hospital, Oromia, Southwest Ethiopia, 2019. BMC Nurs.

[10] Pajnkihar M, Kocbek P, Musović K, Tao Y, Kasimovskaya N, Štiglic G (2020). An international cross-cultural study of nursing students’ perceptions of caring. Nurse Educ Today.

[11] Li M, Zhu WJ, Luo Q, Chen H, Duan Y, Xie HZ. Psychological Experience of Humanistic Care Among Medical Staff in Stroke Wards: A Qualitative Research Study Conducted in China. Frontiers in psychiatry. 2022;13. 10.3389/fpsyt.2022.791993 PMC8989731 35401272

[12] Deng J, Lei L, Zhang H-L, Luo Y (2019). The current status and the influencing factors of humanistic care ability among a group of medical professionals in Western China. Technol Health Care.

[13] Lee LC, Chen CH, Yang YO (2010). The affect of gender on caregiving: a study of male nurses. J Nurs.

[14] Harding T, Jamieson I, Withington J, Hudson D, Dixon A (2018). Attracting men to nursing: is graduate entry an answer?. Nurse Educ Pract.

[15] Shen A, Wang Y, Qiang W (2020). A Multicenter Investigation of Caring Behaviors and Burnout among Oncology Nurses in China. Cancer Nurs.

[16] Erol F, Turk G (2019). Assessing the caring behaviours and occupational professional attitudes of nurses. JPMA The Journal of the Pakistan Medical Association.

[17] Babapour A-R, Gahassab-Mozaffari N, Fathnezhad-Kazemi A. Nurses’ job stress and its impact on quality of life and caring behaviors: a cross-sectional study. BMC Nursing. 2022;21(1). 10.1186/s12912-022-00852-y PMC8968092 35361204

[18] Inocian EP, Cruz JP, Saeed Alshehry A, Alshamlani Y, Ignacio EH, Tumala RB. Professional quality of life and caring behaviours among clinical nurses during the COVID-19 pandemic. Journal of Clinical Nursing. 2021. 10.1111/jocn.15937 PMC8446991 34231269

[19] Fradelos E, Alexandropoulou C-A, Kontopoulou L, Papathanasiou IV, Tzavella F (2022). Factors affecting greek nurses’ caring behaviors: the role of Nurses’ spirituality and the spiritual climate of hospitals. J Relig Health.

[20] Abuhammad S, AlAzzam M, Mukattash T (2021). The perception of nurses towards their roles during the COVID-19 pandemic. Int J Clin Pract.

[21] Prasun MA, Blakeman JR, Vuckovic K, Kim M, Albert N, Stamp KD (2022). Perceptions of changes in practice patterns and patient care among heart failure nurses during the COVID-19 pandemic. Heart & Lung.

[22] Galanis P, Vraka I, Fragkou D, Bilali A, Kaitelidou D (2021). Nurses’ burnout and associated risk factors during the COVID-19 pandemic: a systematic review and meta-analysis. J Adv Nurs.

[23] Li Z, Zuo Q, Cheng J, Zhou Y, Li Y, Zhu L (2021). Coronavirus disease 2019 pandemic promotes the sense of professional identity among nurses. Nurs Outlook.

[24] Sheng Q, Zhang X, Wang X, Cai C (2020). The influence of experiences of involvement in the COVID-19 rescue task on the professional identity among chinese nurses: a qualitative study. J Nurs Adm Manag.

[25] National Bureau of Statistics of China. Statistical Communiqué of the People’s Republic of China on the 2019 National Economic and Social Development 2020 [Available from: http://www.stats.gov.cn/english/PressRelease/202002/t20200228_1728917.html.

[26] Taherdoost H (2017). Determining sample size; how to calculate survey sample size. Int J Econ Manag.

[27] Vasileiou K, Barnett J, Thorpe S, Young T (2018). Characterising and justifying sample size sufficiency in interview-based studies: systematic analysis of qualitative health research over a 15-year period. BMC Med Res Methodol.

[28] Watson R, Lea A (1997). The caring dimensions inventory (CDI): content validity, reliability and scaling. J Adv Nurs.

[29] Tong LK, Zhu MX, Wang SC, Cheong PL, Van IK. A Chinese Version of the Caring Dimensions Inventory: Reliability and Validity Assessment. Int J Environ Res Public Health. 2021;18(13). 10.3390/ijerph18136834 PMC8297071 34202158

[30] Thompson CG, Kim RS, Aloe AM, Becker BJ (2017). Extracting the Variance inflation factor and other Multicollinearity Diagnostics from typical regression results. Basic Appl Soc Psychol.

[31] Ritchie J, Spencer L, Bryman A, Burgess RG (2002). Qualitative data analysis for applied policy research. Analyzing qualitative data.

[32] Allande-Cussó R, Fernández-García E, Gómez-Salgado J, Porcel-Gálvez AM (2022). Understanding the nurse-patient relationship: a predictive approach to caring interaction. Collegian.

[33] Thomas D, Newcomb P, Fusco P (2019). Perception of caring among patients and nurses. J patient experience.

[34] Wei H, Wei T, Brown KJ, Buck SH, Mill MR (2018). Parents’ perceptions of caring characteristics of physicians and nurses. Int J Hum Caring.

[35] Strandås M, Bondas T (2018). The nurse–patient relationship as a story of health enhancement in community care: a meta-ethnography. J Adv Nurs.

[36] Leininger MM (1988). Leininger’s theory of nursing: cultural care diversity and universality. Nurs Sci Q.

[37] Merchant K. How men and women differ: gender differences in communication styles, influence tactics, and leadership styles. Claremont Colleges; 2012.

[38] Street RL (2002). Gender differences in health care provider–patient communication: are they due to style, stereotypes, or accommodation?. Patient Educ Couns.

[39] Al Maqbali M, Al Sinani M, Al-Lenjawi B. Prevalence of stress, depression, anxiety and sleep disturbance among nurses during the COVID-19 pandemic: A systematic review and meta-analysis. Journal of Psychosomatic Research. 2020:110343. 10.1016/j.jpsychores.2020.110343 PMC7831768 33360329

[40] Gómez-Ochoa SA, Franco OH, Rojas LZ, Raguindin PF, Roa-Díaz ZM, Wyssmann BM (2021). COVID-19 in health-care workers: a living systematic review and meta-analysis of prevalence, risk factors, clinical characteristics, and outcomes. Am J Epidemiol.

[41] Wu AW (2020). Health worker well-being and resilience: a Red Ball issue for the COVID-19 response. J Patient Saf Risk Manage.

[42] Barello S, Graffigna G (2020). Caring for Health Professionals in the COVID-19 pandemic emergency: toward an “Epidemic of Empathy” in Healthcare. Front Psychol.

[43] The Lancet (2020). COVID-19: protecting health-care workers. Lancet.

[44] McDougall RJ, Gillam L, Ko D, Holmes I, Delany C (2021). Balancing health worker well-being and duty to care: an ethical approach to staff safety in COVID-19 and beyond. J Med Ethics.

[45] Shalaby SA, Janbi NF, Mohammed KK, Al-harthi KM (2018). Assessing the caring behaviors of critical care nurses. J Nurs Educ Pract.

[46] He J, Liu L, Chen X, Qi B, Liu Y, Zhang Y (2021). The experiences of nurses infected with COVID-19 in Wuhan, China: a qualitative study. J Nurs Manag.

[47] Albougami AS (2020). Perception of caring among nurses working in a Tertiary Hospital in Riyadh, Saudi Arabia. Pak J Med Sci.

[48] Aupia A, Lee T-T, Liu C-Y, Wu S-FV, Mills ME (2018). Caring behavior perceived by nurses, patients and nursing students in Indonesia. J Prof Nurs.

[49] National Health Commission of China (2018). China health yearly statistics 2018.

[50] Mao A, Cheong PL, Van IK, Tam HL (2021). I am called girl, but that doesn’t matter” -perspectives of male nurses regarding gender-related advantages and disadvantages in professional development. BMC Nurs.

[51] Tong LK, Au ML, Li YY, Ng WI, Wang SC (2023). The mediating effect of critical thinking between interest in learning and caring among nursing students: a cross-sectional study. BMC Nurs.

[52] Enns CL, Sawatzky J-AV (2016). Emergency nurses’ perspectives: factors affecting caring. J Emerg Nurs.

[53] Hoeve Yt, Jansen G, Roodbol P. The nursing profession: public image, self-concept and professional identity. A discussion paper. Journal of Advanced Nursing. 2014;70(2):295–309. 10.1111/jan.12177 23711235

[54] Wu LT, Low MMJ, Tan KK, Lopez V, Liaw SY (2015). Why not nursing? A systematic review of factors influencing career choice among healthcare students. Int Nurs Rev.

[55] Romem P, Anson O (2005). Israeli men in nursing: social and personal motives. J Nurs Manag.

[56] Zhang M, zhang P, Liu Y, Wang H, Hu K, Du M (2021). Influence of perceived stress and workload on work engagement in front-line nurses during COVID-19 pandemic. J Clin Nurs.

[57] Wu X, Hayter M, Lee AJ, Yuan Y, Li S, Bi Y (2020). Positive spiritual climate supports transformational leadership as means to reduce nursing burnout and intent to leave. J Nurs Adm Manag.

